# 改进的QuEChERS结合超高效液相色谱-串联质谱法快速测定地表水中双酚类物质

**DOI:** 10.3724/SP.J.1123.2021.03010

**Published:** 2022-01-08

**Authors:** Xuerong TAN, Bin ZHAO, Jianwei LU, Shaoying LIU, Weini GOU, Rong YANG, Peng ZUO

**Affiliations:** 1.广元市疾病预防控制中心, 四川 广元 628000; 1. Guangyuan Center for Disease Control and Prevention, Guangyuan 628000, China; 2.杭州市疾病预防控制中心, 浙江 杭州 310006; 2. Hangzhou Center for Disease Control and Prevention, Hangzhou 310006, China

**Keywords:** 超高效液相色谱-串联质谱, QuEChERS, 双酚类物质, 地表水, ultra performance liquid chromatography-tandem mass spectrometry (UPLC-MS/MS), QuEChERS, bisphenols (BPs), surface water

## Abstract

建立了同时测定地表水中8种双酚类物质(BPs)的超高效液相色谱-串联质谱(UPLC-MS/MS)分析方法。方法基于QuEChERS处理技术,选用乙酸乙酯为提取剂,从基质效应(ME)和萃取回收率(RE)两方面对过程效率(PE)进行优化,确定了50 mg *N*-丙基乙二胺(PSA)和50 mg石墨化炭黑(GCB)混合吸附剂为净化剂。8种BPs使用Waters ACQUITY UPLC BEH C_18_色谱柱(100 mm×2.1 mm, 1.7 μm)进行分离,以甲醇和0.1 mmol/L碳酸氢铵水溶液为流动相进行梯度洗脱,在电喷雾电离(ESI)源、负离子扫描及多反应监测(MRM)模式下进行质谱监测,基质匹配外标法定量。结果表明,8种BPs在8 min内完成色谱分离分析,在各自线性范围内线性关系良好,决定系数(*R*^2^)均大于0.9990,检出限(LOD)和定量限(LOQ)分别为0.1~2.3 ng/L和0.3~6.1 ng/L;以河水为样品基质,在不同添加水平下,8种BPs的平均加标回收率为78.8%~116.6%,相对标准偏差(RSD)为1.8%~9.0%。将该方法用于分析嘉陵江广元段及其支流中BPs的污染状况,结果显示,双酚A(BPA)检出率100%,含量为6.15 ~ 90.03 ng/L;双酚S(BPS)检出率91%,含量为未检出(nd)~4.63 ng/L。研究建立的方法实现了BPs在水中的快速富集与净化,具有简便、快速、灵敏度高和成本低廉等优势,可用于湖泊、河流等地表水中痕量BPs的快速检测。该方法的建立为我国地表水中BPs检测标准的制订和水质安全监控提供了参考。

## Abstract (en)

Chromatography combined with mass spectrometry is the most commonly used detection technology, and it offers the advantages of high sensitivity and high selectivity. However, the contents of bisphenols (BPs) in water samples are at the ng/L level. Even if the BPs contents are determined by mass spectrometry, the samples must be pretreated and enriched. A quick, easy, inexpensive, effective, rugged, and safe technique (QuEChERS) can offer advantages such as cost effectiveness and time efficiency. The application of QuEChERS is currently extended to the analysis of contaminants in food and water samples. In this work, QuEChERS coupled with ultra performance liquid chromatography-tandem mass spectrometry (UPLC-MS/MS) was proposed for the determination of eight BPs in surface water. Method optimization was carried out in terms of process efficiency (PE), which included the matrix effect (ME) and extraction recovery (RE). First, the water samples were extracted with 10 mL ethyl acetate and cleaned with 50 mg primary secondary amine (PSA) and 50 mg graphitized carbon black (GCB). Then, the organic layer was collected and evaporated to dryness under nitrogen flow (40 ℃). Finally, the extracts were redissolved in 0.5 mL of a methanol-water (1:1, v/v) mixture and subjected to UPLC-MS/MS analysis. Chromatographic separation was accomplished on a Waters ACQUITY UPLC BEH C_18_ column (100 mm×2.1 mm, 1.7 μm) with gradient elution. Methanol and 0.1 mmol/L ammonium bicarbonate aqueous solution were used as the mobile phases. The eight BPs were analyzed by MS/MS with negative electrospray ionization (ESI^-^) and in the multiple reaction monitoring (MRM) mode. The matrix matching external standard method was used for quantitative determination. The eight compounds could be analyzed within 8 min. Under the optimized conditions, the calibration curves showed good linearities for the eight BPs, and the coefficients of determination (*R*^2^) were greater than 0.9990. The limits of detection (LODs, *S/N*=3) and limits of quantification (LOQs, *S/N*=10) were in the ranges of 0.1-2.3 ng/L and 0.3-6.1 ng/L, respectively. The average recoveries of the eight BPs in river water samples were 78.8%-116.6%, and the corresponding relative standard deviations (RSDs) were 1.8%-9.0% (*n*=6). This method was applied to the analysis of BPs pollution in Guangyuan section of Jialing river and its tributaries, and widespread pollution was confirmed. The detection rate of BPA was 100% with a content of 6.15-90.03 ng/L, and the detection rate of BPS was 91%, with the contents being in the range not detected (nd) to 4.63 ng/L. The method established in this study allowed for the rapid enrichment and purification of the eight BPs in water. With its advantages of simplicity, rapidness, high sensitivity, and low cost, the method can be used for the rapid determination of trace BPs in surface water such as lake water and river water.

环境内分泌干扰物是一类外源性化学物质,由于其可通过干扰生物或人体内保持自身平衡和调节发育过程天然激素的合成、分泌、运输、结合、反应和代谢等过程,从而对生物或人体的生殖、神经和免疫系统等的功能产生影响,近年来被科学界广泛关注^[1,2]^。双酚类物质(bisphenols, BPs)是典型的环境内分泌干扰物,主要包含双酚A(BPA)、双酚S(BPS)、双酚Z(BPZ)、双酚P(BPP)、双酚AF(BPAF)、双酚F(BPF)、双酚B(BPB)和双酚AP(BPAP)等^[3]^。

BPs是重要的有机化工原料,用于合成聚碳酸酯、环氧树脂、聚砜树脂等高分子材料,在涂料、膜材料、光电科技、包装材料及其他日用品领域被广泛应用^[4,5]^。在这些产品制造、使用、老化和处置过程中,BPs会释放到环境中,对人体产生危害。BPs具有雌激素效应,能与多种内源性受体相互作用,如甲状腺激素^[6,7]^、糖皮质激素^[8]^、雄激素和雌激素受体^[9,10,11]^,对人体健康构成严重威胁。报道^[12,13,14,15]^还表明,BPs对哺乳动物和鱼类等均具有明显的神经毒性,能够影响神经功能及神经递质含量等。近年来,世界各地的环境基质^[16,17]^、食品基质^[18]^、饮用水^[19]^和人体生物样本^[20]^中均有BPs被检出的报道。我国多个河流被报道检测出双酚A,龚剑等^[21]^检测出珠江广州河段表层水中双酚A的含量为97.8~540.6 ng/L, Bian等^[22]^对长江河口和东海入海口进行了双酚A的测定,发现在上层底泥中其含量为0.7~13.2 ng/g。

BPs主要检测方法包括液相色谱法^[23]^、液相色谱-质谱法^[24]^、气相色谱-质谱法^[25]^等。随着居民饮食安全意识的提高和对水中BPs污染状况的关注,建立水中BPs的高灵敏度、高选择性、高通量检测分析方法变得越来越迫切。色谱与质谱技术联用具有高灵敏度及高选择性等优点,是最常用的检测技术。但水样中BPs的含量处于ng/L水平,需要对样品进行前处理,以达到富集和净化的目的^[26]^。传统的液液萃取耗费大量有机溶剂,且耗时长,灵敏度低;固相萃取是最常用的前处理方式,具有纯化效果好、浓缩倍数大、重现性好、自动化操作程度高等优点,但处理步骤较复杂,成本高。QuEChERS方法简单,试剂用量少,样品损失少,回收率高,成本低廉。

本研究基于QuEChERS前处理技术,选用乙酸乙酯为提取剂,*N*-丙基乙二胺(PSA)和石墨化炭黑(GCB)为净化剂,结合超高效液相色谱-串联质谱(UPLC-MS/MS)技术,实现了地表水中8种BPs的快速定性和定量。该方法具有简便、快捷、灵敏度高和成本低廉等优势,能快速富集与净化水中BPs。最后,运用此方法检测嘉陵江广元段及其支流中BPs的污染状况,证明该方法适用于地表水中8种BPs的痕量分析。

## 1 实验部分

### 1.1 仪器与试剂

QTRAP 4500超高效液相色谱-串联质谱仪(美国AB Sciex公司); Milli-Q超纯水系统(美国Millipore公司); HAC-24A氮吹浓缩仪(天津市恒奥科技公司); HR/T20MM高速冷冻离心机(湖南赫西仪器装备有限公司)。双酚A(纯度98%)、双酚S(纯度98%)、双酚Z(纯度99%)、六氟双酚A(纯度95%)、双酚F(纯度95%)、双酚B(纯度95%)和双酚AP(纯度95%)均购自德国Dr. Ehrenstorfer公司;双酚P(纯度95%)、乙酸(分析纯)、无水硫酸镁(MgSO_4_,分析纯)和乙酸钠(NaAC,分析纯)均购自美国Sigma-Aldrich公司;PSA、GCB和C_18_均购自美国Agilent公司;甲醇、乙腈和乙酸乙酯均为农残级,购自美国J. T. Baker公司。

准确称取8种BPs各100 mg,分别用甲醇溶解并定容至100 mL,配制成质量浓度均为1 mg/mL的标准储备液,于-20 ℃保存。

分别移取适量上述8种单标准储备液,用甲醇配制混合标准中间液,其中BPA、BPZ、BPAP和BPAF质量浓度为50 mg/L, BPF、BPP和BPB质量浓度为100 mg/L, BPS质量浓度为10 mg/L;用甲醇-水(1∶1, v/v)对上述混合标准中间液进行逐级稀释,配制成系列混合标准工作溶液。

### 1.2 实验方法

1.2.1 样品采集

根据HJ/T 91-2002,对广元市境内嘉陵江、白龙江、南河3条河流分上、中、下游3个断面监测布点。嘉陵江上游断面设在广元市朝天区大滩镇小河口,断面编号J1;中游断面设在广元市利州区澳援大桥,断面编号J2;下游断面设在苍溪县陵江镇百利村,断面编号J3。白龙江上游断面设在广元市青川县姚渡大桥,断面编号B1;白龙江中游断面设在广元市利州区三堆镇,断面编号B2;白龙江下游断面设在广元市昭化区两河口,断面编号B3。南河上游断面设在广元市利州区和平村,断面编号N1;中游断面设在广元市利州区龙洞碥大桥,断面编号N2;下游断面设在利州区天成大桥,断面编号N3,详见图1。各横断面设置6个采样点(两岸对称设点,每个采样点间隔50 m),使用不锈钢采水器采集0.5 L表层河水(距离河岸1.5 m,河面下0.5 m),共采集54份,分别装入玻璃瓶中,冷藏运输,保存于4 ℃冰箱,待测。

**图 1:**
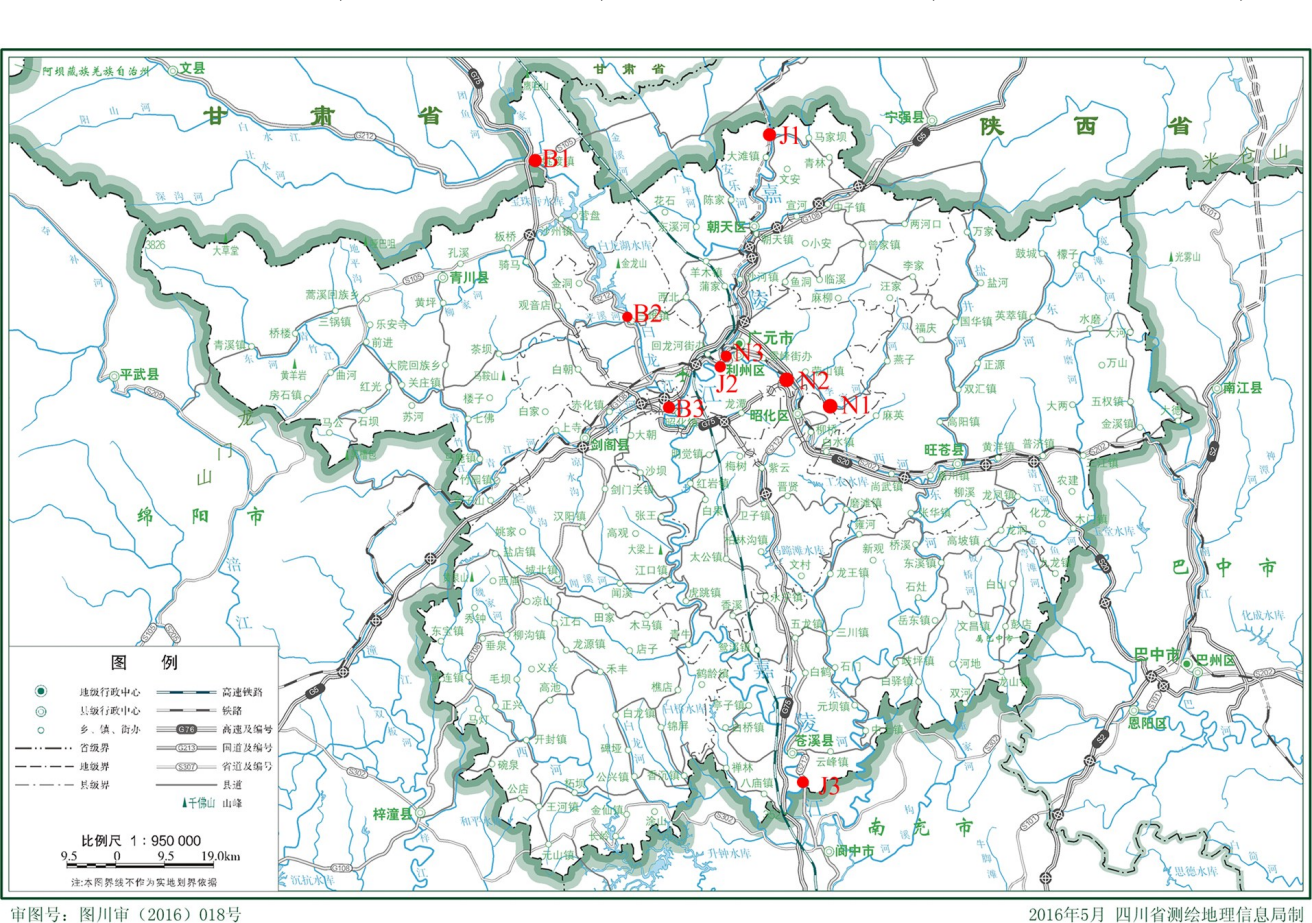
河流采样断面分布图

1.2.2 样品前处理

移取40 mL水样,置于50 mL比色管中,加入10 mL乙酸乙酯,剧烈摇荡,提取10 min,静置5 min,取有机层于15 mL聚丙烯(PP)离心管中,加入50 mg PSA和50 mg GCB净化,以10000 r/min离心5 min,取上清液于45 ℃下氮气吹干,用0.5 mL甲醇-水(1∶1, v/v)溶解残渣,待UPLC-MS/MS测定。

1.2.3 分析条件

色谱柱Waters ACQUITY UPLC BEH C_18_柱(100 mm×2.1 mm, 1.7 μm);柱温40 ℃;流动相A甲醇;流动相B 0.1 mmol/L碳酸氢铵水溶液;流速0.3 mL/min。梯度洗脱条件:0~0.5 min, 30%A; 0.5~4.0 min, 30%A~95%A; 4.0~6.0 min, 95%A; 6.0~7.0 min, 95%A~30%A; 7.0~8.0 min, 30%A。进样体积5 μL。

电喷雾电离(ESI)源,负离子扫描模式;离子源温度600 ℃;离子化电压(IS), -4500 V;去簇电压-85.0 V;气帘气(CUR)压力172.4 Pa;喷雾气(GS1)压力379.2 Pa;辅助加热气(GS2)压力379.2 Pa;多反应监测(MRM)模式。8种BPs的保留时间和质谱参数见表1。

**表 1:** MRM模式下8种BPs的保留时间与质谱参数

Analyte	Retention time/min	Precursor ion (m/z)	Product ions (m/z)	Declustering potential/V	Collision energies/eV
Bisphenol S (BPS)	1.94	249.0	108.0^*^, 155.8	-80	-34, -30
Bisphenol F (BPF)	4.10	199.0	76., 92.6	-80	-35, -27
Bisphenol A (BPA)	4.64	227.0	212.0^*^, 133.2	-80	-25, -36
Bisphenol B (BPB)	4.91	241.0	210.6^*^, 147.0	-90	-36, -36
Bisphenol AF (BPAF)	5.00	335.0	264.8^*^, 68.7	-110	-30, -50
Bisphenol AP (BPAP)	5.05	289.0	273.8^*^, 194.8	-95	-26, -35
Bisphenol Z (BPZ)	5.21	267.0	172.7^*^, 144.7	-110	-39, -45
Bisphenol P (BPP)	5.62	345.0	315.0^*^, 132.8	-110	-50, -47

* Quantitative ion.

## 2 结果与讨论

### 2.1 LC-MS/MS条件的优化

取500 ng/mL 8种目标物的标准溶液,分别以自动进样的方式在全扫检测模式下进行质谱条件优化。结果表明,8种BPs在ESI^-^模式下得到的[M-H]^-^离子峰的响应值更佳。通过子离子扫描得到目标物碎片离子信息,选择响应最强的产物离子和次强的产物离子分别作为8种BPs的定量离子(quantitative ion)和定性离子(qualitative ion),并对去簇电压和碰撞能量进行优化,优化结果见表1。

取8种目标物的混合标准溶液,以甲醇和水为流动相,按1.2.3节梯度洗脱程序,在保持浓度、流速、进样量等参数一致的条件下,分别考察了用亚乙基桥杂化颗粒填充的Waters ACQUITY UPLC BEH C_18_色谱柱(100 mm×2.1 mm, 1.7 μm)和用高强度硅胶颗粒填充的Waters ACQUITY HSS T_3_色谱柱(100 mm×2.1 mm, 1.7 μm)对目标物色谱峰形、离子化效率和保留时间的影响(见图2)。结果表明,BPF、BPAF、BPAP和BPZ在C_18_柱上能获得更好的峰形和检测灵敏度,故选择C_18_柱为分析柱。

**图 2:**
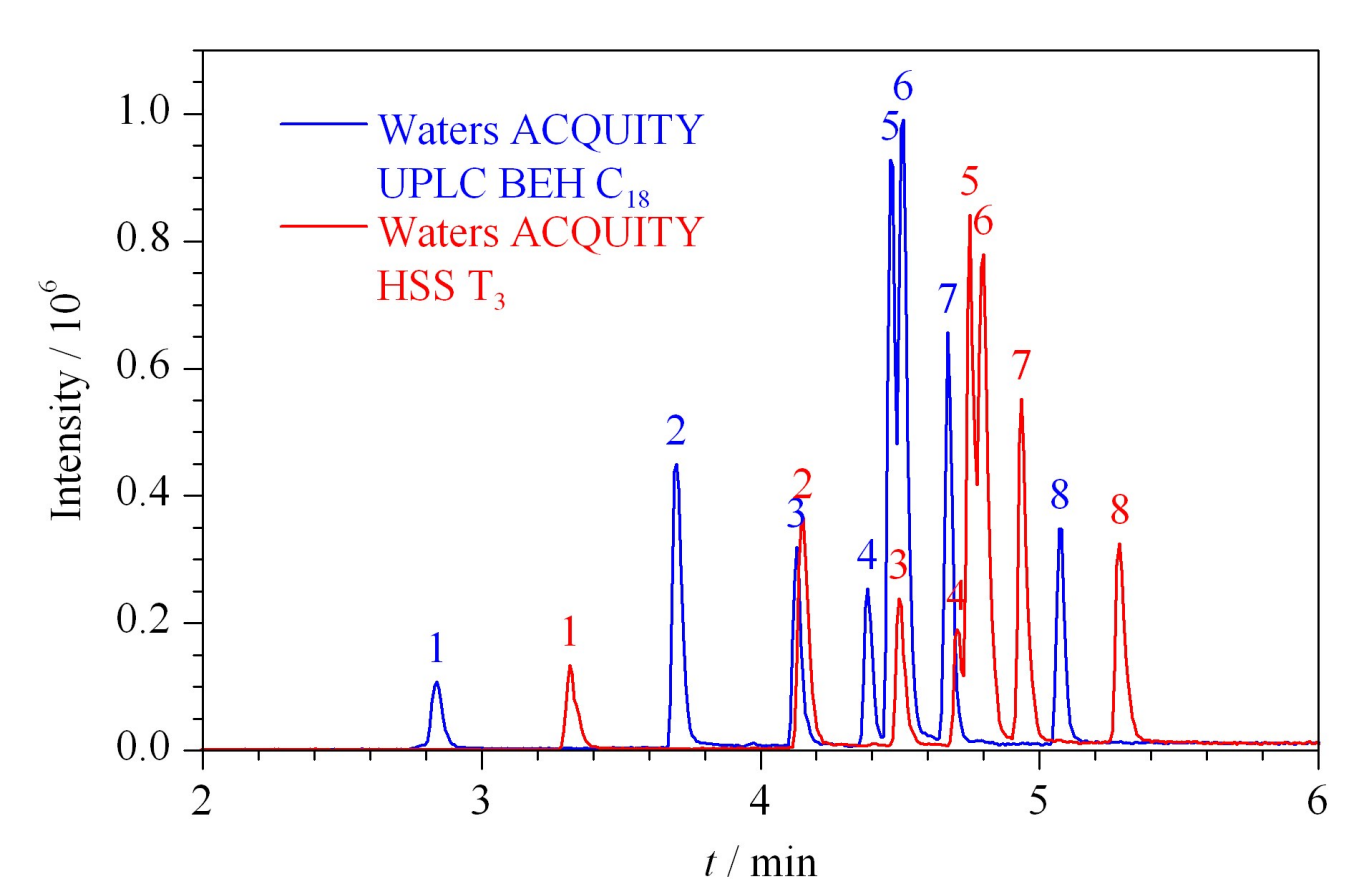
采用不同色谱柱时8种BPs混合标准溶液的总离子流色谱图

实验同时还对流动相种类、流速、柱温及梯度洗脱程序进行了优化,最终采用甲醇和0.1 mmol/L碳酸氢铵水溶液作为流动相。流动相中加入碳酸氢铵有助于化合物的离子化,使目标物响应增大,提高灵敏度。在最优条件下得到的8种目标化合物的总离子流色谱图见图3,可以看出,8种BPs在8 min内可较好分离。

**图 3:**
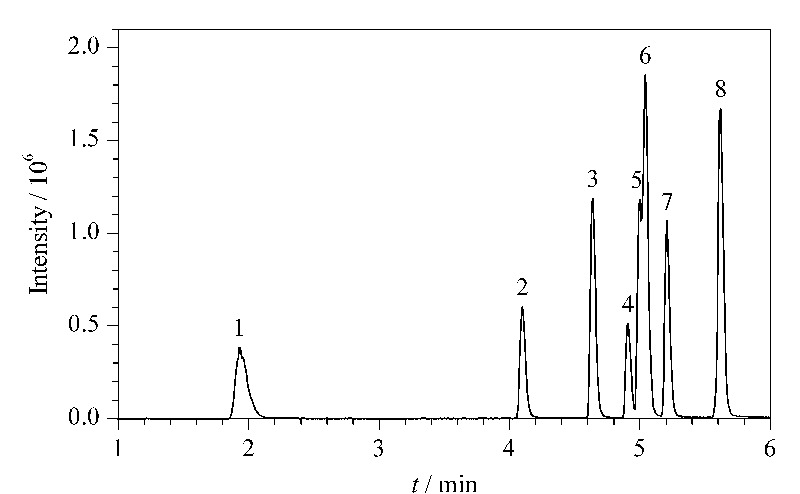
8种BPs混合标准溶液的总离子流色谱图

### 2.2 QuEChERS方法的优化

本研究选用乙酸乙酯作为提取溶剂,将8种BPs混合标准溶液加入到40 mL河水中,使得BPA、BPZ、BPAP和BPAF质量浓度为2.5 ng/mL, BPF、BPP和BPB质量浓度为5.0 ng/mL, BPS质量浓度为0.5 ng/mL,以此为例优化QuEChERS方法。每次试验均平行3次,以检验方法的重复性。

2.2.1 提取液的优化

实验在保持其他条件一致的情况下,以1 g NaAC+4 g MgSO_4_为萃取盐,比较了含1%(v/v)甲酸的乙酸乙酯溶液、含1%(v/v)乙酸的乙酸乙酯溶液,以及纯乙酸乙酯对提取效果的影响。结果表明,是否加酸对8种目标物的提取效果影响不明显,故本研究中提取液不加酸,用乙酸乙酯提取。

实验在保持其他条件一致的情况下,采用乙酸乙酯提取,以不加盐的空白组为对照,考察5 g NaAC、1 g NaAC+4 g MgSO_4_和5 g MgSO_4_萃取盐对提取效果的影响。结果表明,与加盐的组别相比,不加盐的对照组8种BPs的响应值更高(见图4)。故本研究中提取液不加盐。

**图 4:**
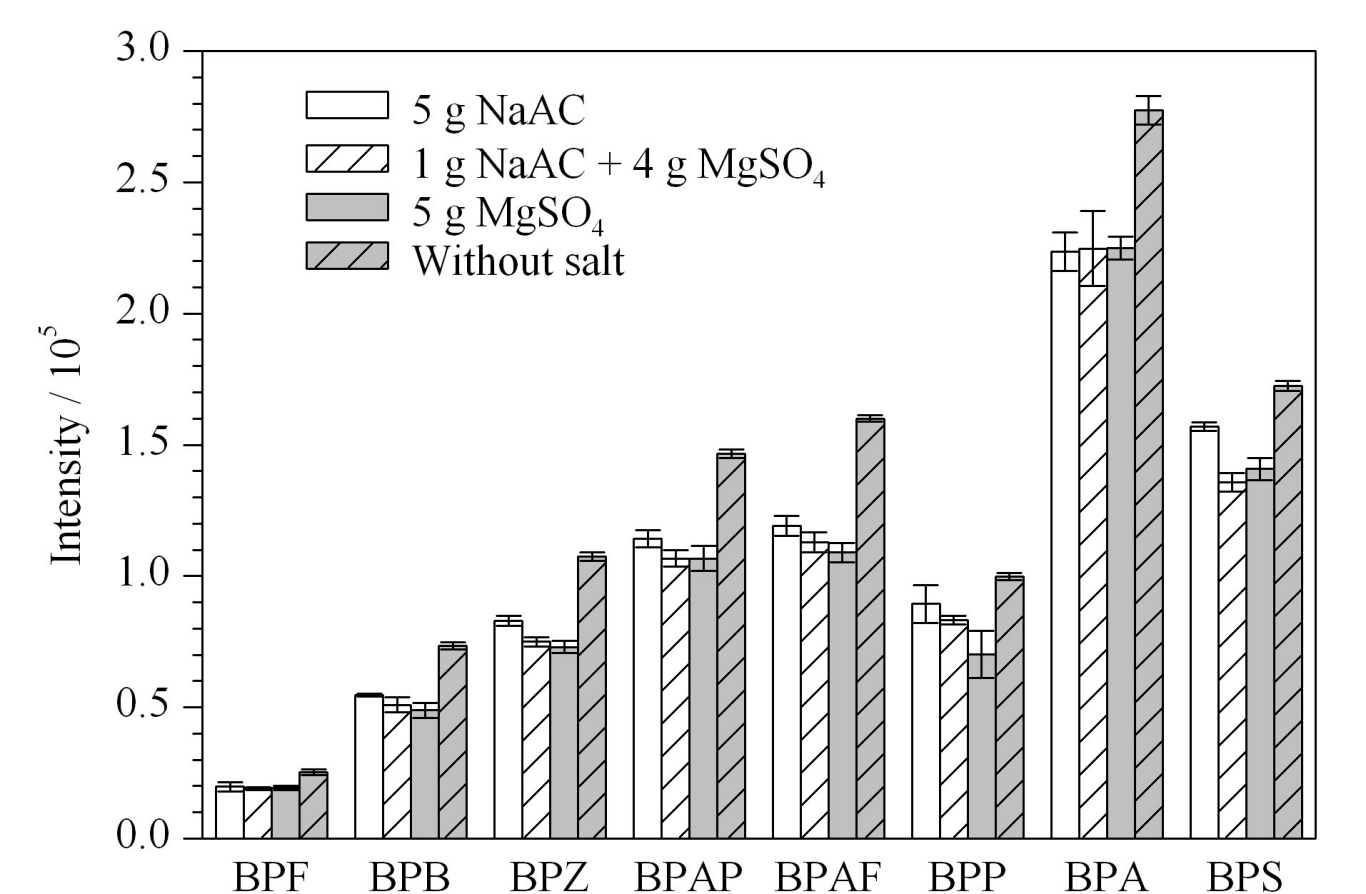
不同萃取盐对8种BPs响应强度的影响(*n*=3)

2.2.2 净化剂的优化

提取方法的优化以过程效率(process efficiency, PE)最大化为基础,它考虑了样品处理过程中的基质效应(matrix effect, ME),评估了样品处理程序中分析物损失对分析物信号的影响,即回收效率(recovery efficiency, RE)。其中,PE=RE×ME×100%; RE=*C*_1_/*C*_2_×100%; ME=*C*_2_/*C*_3_×100%(*C*_1_为样品加入标准物质并经程序处理得到的峰面积,*C*_2_为样品经程序处理之后加入标准物质得到的峰面积,*C*_3_为空白样品经程序处理之后加入标准得到的峰面积)^[27,28]^。ME评价基质对被分析物信号的影响,包含3种情况:ME=100%,没有任何影响;ME>100%,增强被分析物信号;ME<100%,抑制被分析物信号。

按照样品前处理过程,以无吸附剂的空白组为对照,分别选取PSA、C_18_和GCB为净化剂,比较不同吸附剂组合对RE、ME和PE的影响,结果见图5。

**图 5:**
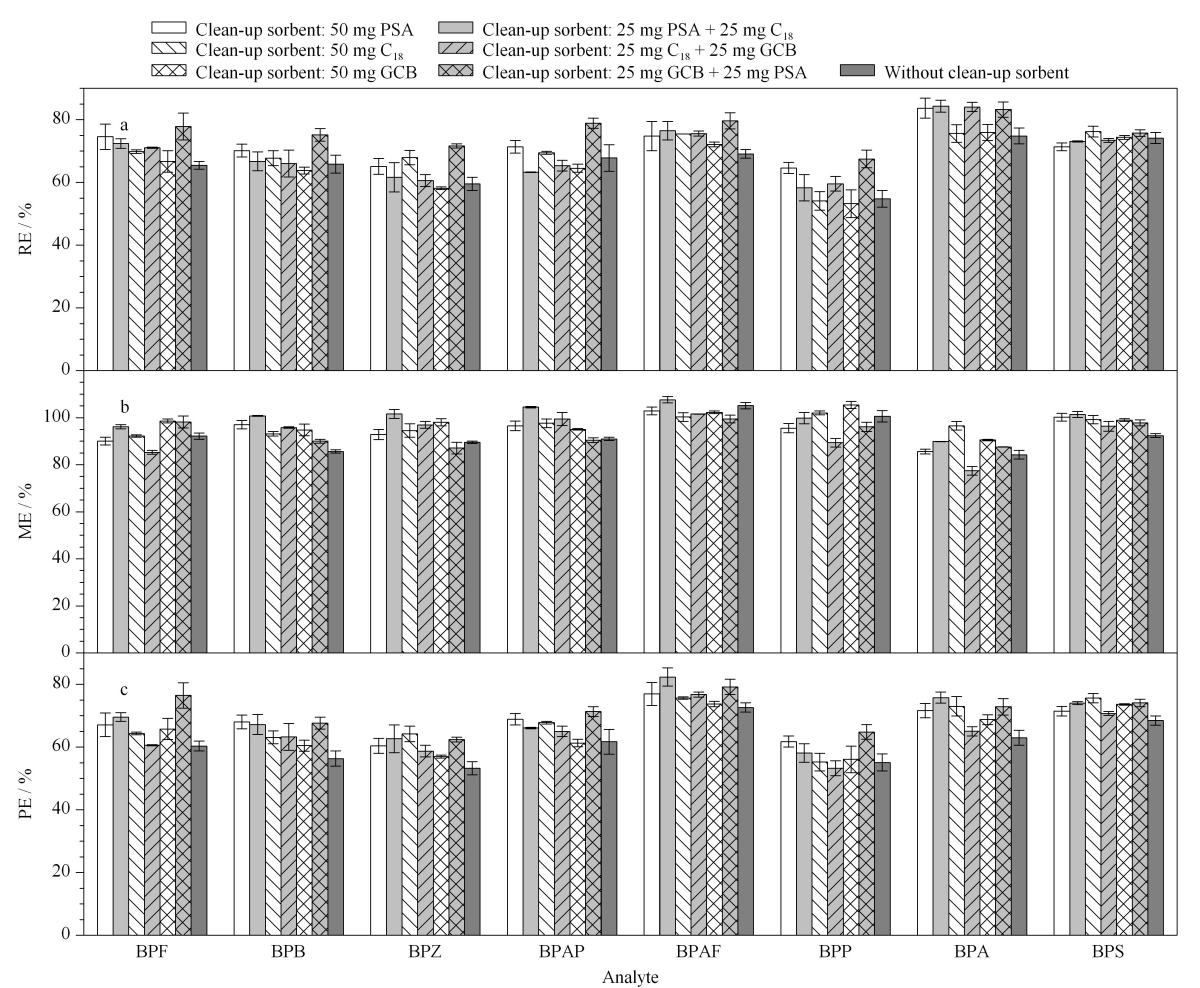
不同吸附剂对8种BPs(a)RE、(b)ME和(c)PE值的影响(*n*=3)

图5的结果显示,不同种类的吸附剂对8种BPs的RE影响明显,如仅使用GCB时,BPZ的RE为58.1%,但当使用GCB+PSA时,其RE升高至71.6%。不同种类的吸附剂对目标分析物的ME影响相对较小,大部分集中在90%~100%,以离子抑制效应为主导。与对照组相比,大部分的吸附剂都能提高目标分析物的RE和ME值。因此,为了提高样品处理的回收效率,不可避免地需要净化步骤。提取方法的优化以过程效率PE最大化为最终目标,从图5中可以看到,GCB+PSA为最优组合,所以选择GCB+PSA混合吸附剂作为本方法的净化剂。

选取25 mg GCB+25 mg PSA、50 mg GCB+50 mg PSA和100 mg GCB+100 mg PSA对净化剂用量进行优化。随着净化剂用量的增加,部分分析物的PE值被提高,但趋势不明显。综合考虑8种目标物的PE值,最终选择50 mg GCB+50 mg PSA作为本研究的吸附剂。

### 2.3 方法学考察

2.3.1 线性范围与检出限

取7份空白河水,加入系列混合标准溶液,按照1.2.2节和1.2.3节步骤处理,分别以8种BPs的质量浓度为横坐标,以峰面积为纵坐标,绘制目标分析物的基质加标校正曲线(见表2)。结果显示,8种BPs在各自范围内均能获得良好的线性关系,决定系数(*R*^2^)均大于0.9990。以信噪比(*S/N*)=3和10确定方法的检出限(LOD)和定量限(LOQ)。8种BPs的LOD为0.1~2.3 ng/L, LOQ为0.3~6.1 ng/L。

**表 2:** 8种BPs的线性范围、线性方程、决定系数、检出限和定量限

Analyte	Linear range/(ng/mL)	Linear equation	R^2^	LOD/(ng/L)	LOQ/(ng/L)
BPS	0.05-4.00	Y=2.11×10^5^ X-6.96×10^2^	0.9996	0.1	0.3
BPF	0.50-40.00	Y=1.74×10^4^ X-5.06×10^2^	0.9997	2.3	6.1
BPA	0.25-20.00	Y=5.81×10^4^ X+1.20×10^2^	0.9997	0.3	1.0
BPB	0.50-40.00	Y=1.46×10^4^ X-2.38×10^2^	0.9990	1.9	5.9
BPAF	0.25-20.00	Y=7.44×10^4^ X-3.72×10	0.9999	0.5	1.5
BPAP	0.25-20.00	Y=1.04×10^5^ X-2.95×10^2^	0.9999	0.4	1.5
BPZ	0.25-20.00	Y=4.41×10^4^ X-1.01×10^2^	0.9997	0.5	1.8
BPP	0.50-40.00	Y=3.75×10^4^X-5.85×10^2^	0.9997	0.5	1.8

Y: peak area; X: mass concentration, ng/mL.

2.3.2 回收率和精密度

取河水样品40 mL,做3个水平的加标回收试验,每个添加水平做6次平行,计算各待测物的平均回收率和相对标准偏差(RSD),结果见表3。方法的平均加标回收率为78.8%~116.6%, RSD为1.8%~9.0%,准确度和精密度均符合HJ/T 91-2002《地表水和污水监测技术规范》要求。这表明该方法适用于分析湖泊、河水等地表水中8种BPs。

**表 3:** 河水中8种BPs的加标回收率和精密度(n=6)

Analyte	Content/ (ng/L)	Low				Medium				High		
		Added/ (ng/mL)	Recovery/ %	RSD/ %		Added/ (ng/mL)	Recovery/ %	RSD/ %		Added/ (ng/mL)	Recovery/ %	RSD/ %
BPS	<LOQ	0.05	94.9	8.0		0.40	104.6	1.8		2.00	89.6	8.4
BPF	nd	0.50	112.1	3.4		4.00	84.8	9.0		20.00	99.7	4.1
BPA	9.5	0.25	104.0	7.2		2.00	91.0	8.2		10.00	106.9	2.9
BPB	nd	0.50	78.8	8.3		4.00	94.4	4.8		20.00	90.7	2.4
BPAF	nd	0.25	95.0	6.1		2.00	80.0	7.2		10.00	89.0	2.6
BPAP	nd	0.25	116.6	2.7		2.00	84.3	7.1		10.00	98.9	2.4
BPZ	nd	0.25	90.4	7.8		2.00	80.4	7.0		10.00	88.9	3.6
BPP	nd	0.50	98.7	4.4		4.00	80.8	6.3		20.00	92.7	4.1

nd: not detected.

2.3.3 与其他方法比较

表4列出了近两年文献中报道的测定水中BPs的前处理方法,并对各自性能做了简单对比。固相萃取具有纯化效果好、灵敏度高、重现性好等优点,但处理步骤较复杂,成本高,部分化合物回收率低,如Zhang等^[19]^和Huang等^[29]^文献中报道的部分化合物的回收率仅为57%和31%。采用GC-MS和GC-MS/MS分析BPs存在一定的局限性,如Skufca等^[30]^报道的方法,LOQ为10~30 ng/L,灵敏度低;Wang等^[31]^通过固相萃取和柱前衍生相结合的方式提高了方法的检测灵敏度,但操作繁琐。苟新磊等^[32]^利用真空冷冻干燥浓缩水样,操作简单,灵敏度高,回收率好,但方法中未添加净化步骤,只适合基质单一的瓶装水。与以上方法相比,本研究中的QuEChERS方法操作简单,试剂用量少,成本低廉,回收率高,结合UPLC-MS/MS,具有检测灵敏度高、抗干扰能力强、定性准确等优点。

**表 4:** 与其他文献方法的比较

Pretreatment	Detection method	LOQ/(ng/L)		Recovery/%		Ref.
HLB SPE column	UPLC-MS/MS	0.1-	1.7	57-	97	[19]
Oasis HLB SPE cartridges	UPLC-MS/MS	0.04-	5.26	31-	126	[29]
MCX Prime SPE cartridges	GC-MS/MS	10-	30	78-	106	[30]
Oasis HLB SPE cartridge and derivatization	GC-MS	2.04-	7.67	73-	115	[31]
Freeze dried under vacuum	UPLC-MS/MS	0.04-	3.33	75-	102	[32]
LLE and precolumn derivatization	UPLC-MS/MS	5-	20	81-	119	[33]
QuEChERS	UPLC-MS/MS	0.3-	6.1	78.8-	116.6	this work

LLE: liquid-liquid extraction.

### 2.4 实际样品的测定

通过分析来自嘉陵江广元段及其支流54份地表水中目标化合物的含量,进一步验证分析方法的适用性。实验过程中,每一批样品做3个程序空白实验,计算样品含量时扣除程序空白实验中BPs的含量。

白龙江、嘉陵江和南河均检出BPA和BPS,部分采样点检出微量的BPF、BPP和BPAP,但均低于检出限,详细的结果如表5所示。BPA在白龙江、嘉陵江和南河中的检出率均为100%,其中南河的BPA含量最高(平均含量37.80 ng/L,含量范围10.15 ~ 90.03 ng/L),而南河龙洞碥断面(N2)检出值居首,平均65.14 ng/L。此断面上游有许多的小型工厂,排污口相对较多,且南河水量小,水流速度慢,自净能力差,这可能是导致其BPA含量偏高的原因。BPS在白龙江、嘉陵江和南河中的检出率分别为94%、78%和100%,其中嘉陵江中BPS的含量最高(平均含量1.05 ng/L,含量范围nd~4.63 ng/L),而嘉陵江天成大桥断面(J2)检出值排第一,平均2.64 ng/L。此断面横穿人口稠密的广元老城区,又处在嘉陵江与南河交汇处的下游,生活污水导致BPS含量偏高。白龙江中BPA和BPS的平均含量分别为20.25 ng/L和0.54 ng/L,其远离市区,水量相对充沛,故BPA和BPS含量相对较低。

**表 5:** 白龙江、嘉陵江和南河水样中BPs的含量

Analyte	Bailong River			Jialing River			Nan River		
	Mean	Median	Range	Mean	Median	Range	Mean	Median	Range
BPA	20.25	21.27	6.15-35.42	28.59	31.66	13.28-54.43	37.80	32.96	10.15-90.03
BPS	0.54	0.44	nd-1.25	1.05	0.31	nd-4.63	0.49	0.44	0.25-1.13
BPs	20.78	21.78	6.53-36.78	29.64	32.03	13.28-55.40	38.30	33.40	10.78-90.65

nd: not detected.

3条河水中均检出BPA和BPS,表明广元及其周边地区以BPA的使用为主。BPA在中国是一种高产量的化学品,产量约为16.7万吨/年^[34]^。自2008年,中国开始规范BPA的生产和使用,其逐渐被BPS和BPF所取代。但广元境内河流未发现高浓度的BPS和BPF,仍然以BPA为主,且BPA的水平(6.15~90.03 ng/L)普遍低于其他河流,如辽河流域(4~141 ng/L)^[35]^、松花江(23~714 ng/L)^[36]^、珠江(43~639 ng/L)^[37]^等。这些结果表明BPs在全国不同地区的使用量存在明显差异。

## 3 结论

本文建立了QuEChERS-UPLC-MS/MS检测地表水中8种BPs的分析方法。该方法具有简便、快速、灵敏度高、重复性好、回收率佳和成本低廉等优势,可用于湖泊、河流等地表水中痕量BPs的快速检测,具有实际应用价值。将该方法应用于测定嘉陵江广元段及其支流中BPs的含量,结果显示污染物主要为BPA和BPS,存在影响人体健康的风险,这些结果将为了解BPs在水环境中的污染情况提供有用信息。
